# Using Technology to Facilitate Fidelity Assessments: The Tele-STAR Caregiver Intervention

**DOI:** 10.2196/13599

**Published:** 2019-05-24

**Authors:** Allison Lindauer, Glenise McKenzie, David LaFazia, Loriann McNeill, Kate Mincks, Natasha Spoden, Marcella Myers, Nora Mattek, Linda L Teri

**Affiliations:** 1 Layton Aging and Alzheimer's Disease Center Oregon Health & Science University Portland, OR United States; 2 School of Nursing Oregon Health & Science University Portland, OR United States; 3 School of Social Work University of Washington Seattle, WA United States; 4 Northwest Research Group on Aging School of Nursing University of Washington Seattle, WA United States; 5 Family Caregiver Support Program Multnomah County, Oregon Portland, OR United States; 6 Northwest Roybal Center School of Nursing University of Washington Seattle, WA United States

**Keywords:** dementia, caregiving, fidelity

## Abstract

**Background:**

Families living with Alzheimer disease and related dementias have more access to support thanks to the development of effective telehealth-based programs. However, as technological science grows, so does the risk that these technology-based interventions will diverge from foundational protocols, diluting their efficacy. Strategies that ensure programs are delivered as intended, with fidelity to guiding protocols, are needed across the intervention spectrum—from development to wide-scale implementation. Few papers address fidelity in their technology-based work. Here, we present our translated telehealth intervention, Tele-STAR, with our fidelity findings.

**Objective:**

This study aimed to assess the preliminary efficacy of Tele-STAR on reducing family caregiver burden and depression. Across the implementation phases, we assessed the fidelity of a caregiver education intervention, STAR-C, as it was translated into a telehealth option (Tele-STAR).

**Methods:**

A total of 13 family caregivers consented to participate in an 8-week, videoconference-based intervention (Tele-STAR). Tele-STAR efficacy in reducing the affective burden of caregiving was assessed using pre- and postintervention paired t tests. Content experts assessed program fidelity by reviewing and rating Tele-STAR materials for adherence to the original STAR-C protocol. These experts assessed treatment fidelity by viewing videos of the intervention and rating adherence on a checklist.

**Results:**

Tele-STAR reduced caregiver burden and retained good program and treatment fidelity to STAR-C.

**Conclusions:**

We found Tele-STAR reduced caregiver burden and had good fidelity to the original protocol. Assessing fidelity is a complex process that requires incorporation of these procedures early in the research process. The technology used in this study facilitated the accrual of informative data about the fidelity of our translated intervention, Tele-STAR.

## Introduction

### Background

Caring for one of the world’s 47 million adults with Alzheimer disease or a related dementia can be both rewarding and taxing for family members [1,2]. For some, caregiving can have detrimental effects on their mental health, leading to depression and a sense of burden [3]. The strain of caregiving can also have negative effects on quality of life for the person with dementia. Stressed families are more likely to consider long-term care placement for those with dementia, which may alleviate some of the caregiving tasks but often not the affective symptoms of depression and pre-death grief [4-6].

Interventions such as educational programs and support groups are available to family caregivers for those with dementia. These interventions are often effective [7] but caregivers report access challenges. Distance, cost, neuropsychiatric symptoms of dementia and time constraints all hinder caregiver engagement in these opportunities [8,9]. Real-time, internet-based videoconferencing technology (also known as telehealth) makes education and support interventions accessible for families living with dementia. Here, we report on our work developing and assessing an internet-based intervention to provide accessible support for family members.

Telehealth-based interventions are appealing because caregivers can receive help and support in their own homes. These interventions have small to moderate effects on reducing caregiver burden and depression and good consumer acceptance [10,11]. However, despite evidence that family caregivers prefer individualized interventions with real-time counselors, most telehealth interventions are group-based, automated, and not tailored to stages of disease [11-14].

To address the need for personalized, real-time educational interventions for families caring for those with dementia, we designed Tele-STAR. Tele-STAR uses videoconferencing to connect nurse consultants with family caregivers to guide caregivers in strategies to reduce the emotional, cognitive, and physical effects of distressing behavioral symptoms of dementia. Tele-STAR was developed from the in-person intervention, STAR-C [15]. STAR-C employs cognitive behavioral techniques to increase caregiver awareness of care partner behavior and their reactions to them. The efficacy of the 8-week STAR-C intervention has been established in earlier work [15,16]. STAR-C is implemented face-to-face in families’ homes. However, in rural states such as Oregon, implementation for families outside metropolitan areas requires a different approach [16]. Consequently, we revised the STAR-C intervention into a telehealth option to increase access to anyone with a computer and internet connection.

To preserve the efficacy of STAR-C, we sought to retain its essential components as it was translated into Tele-STAR. Our aims were to (1) explore the preliminary efficacy of Tele-STAR and (2) assess the fidelity of Tele-STAR to the original STAR-C [15], after we converted it from an in-person intervention to a telehealth-based version.

### Fidelity Assessment

Fidelity assessment ascertains if an intervention adheres to prescribed protocols and treatments across the implementation spectrum, from early-stage pilots to full-fledged wide-scale interventions [17,18]. High fidelity to an intervention increases confidence in internal validity so that scientists (and other consumers) can trust that the effects of the intervention resulted from the intervention and not extraneous factors [17]. A fidelity assessment can be used to examine adherence to a novel intervention in the early stages of implementation, to evaluate if a translated program is faithful to the original protocol across the stages of an intervention, or to prevent divergence from the protocol and ensure consistent delivery in large-scale programs [18].

Recognizing the value of fidelity, the US National Institute on Aging encourages scientists to consider fidelity assessments in their behavioral research [19]. Onken et al (2014) [19] argue that behavioral interventions cannot be adequately implemented if a fidelity assessment plan is not in place. Without a fidelity plan, community providers lack guidelines to ensure an intervention is delivered as intended.

We sought to determine if Tele-STAR retained the important components of the original intervention (STAR-C) [15], if the nurse consultants adhered to the intervention protocol, and if caregivers received and enacted the information provided. Thus, we assessed 2 types of fidelity: program and treatment, to evaluate how closely Tele-STAR aligned with STAR-C [15].

Our fidelity assessment, modeled on Teri’s (2010) and Griffiths’ (2016) work, examined both program fidelity and treatment fidelity. A program fidelity assessment examines if a program retains the theoretical foundation and essential components of an intervention when it is translated into a telehealth intervention. Treatment fidelity assessment explores if a treatment is implemented and received as planned, based on the program design [17,20,21].

## Methods

### Participants

We recruited family caregivers from the local community and our dementia clinic. Participants had to provide care at least 4 hours/day for a family member with Alzheimer disease or a related dementia, speak English, and have access to a functional computer. They were not required to live with their family member but all did. We accepted participants from Oregon and Southwest Washington, without regard to their distance from the study center. Caregivers were not paid for their work, nor did they receive any financial compensation for study participation. Each caregiver had to report 3 or more care recipient behaviors that were distressing for them. These standardized questions were embedded in the initial telephone screening interview [15].

Most caregivers owned a computer with internet connectivity (broadband, Wi-Fi, or cellular); we lent a laptop with cellular service to those who did not have a functional connection. All caregivers consented to having the visits recorded. We did not query caregivers about the type of computer they used (eg, computer, laptop, and smartphone), but we did assess the age of their device. We assessed caregivers’ level of comfort and knowledge with computer use.

To further characterize the sample of caregivers, we asked them how often they contacted their health care provider (from once a year or less to more than once a week) and if they attended a support group. We also queried caregivers on how often they contacted their care recipient’s health care provider and how often they gave their care recipient “as needed” medications for behaviors. The computer, health care–usage and satisfaction surveys were all emailed to caregivers, allowing them to send anonymous replies using Qualtrics [22].

All participants provided consent over the telephone. Although the care recipients did not participate in the intervention, we required their consent for their caregiver to participate. If they were unable to consent, we followed Oregon Health and Science University’s (OHSU’s) Decisionally Impaired protocol [23] and sought assent from the family member. All components of this study were approved by OHSU’s Institutional Review Board (IRB# 17526).

### Tele-STAR Procedures

Measures used in previous STAR-C studies [15,16,24] were used in Tele-STAR (Table 1). Caregivers were assessed before, during (after the 4th visit), and after the full 8-week intervention and then 2 months after the intervention. Demographic information was collected before the initiation of the intervention. We asked caregivers to estimate the number of daily hours of caregiving they provided per week. The research assistant administered all the measures in Table 1 via the direct-to-home videoconferencing interface.

A total of 2 consultants (AL and MM) provided the Tele-STAR intervention to the family caregivers over 8 weekly sessions. One consultant is a licensed practical nurse (LPN); the other, the principal investigator (PI), is a PhD-prepared gerontological nurse practitioner (GNP). The GNP, an experienced interventionist, trained the LPN in the Tele-STAR protocol using a training outline developed by the PI. The LPN received instruction in the booklets, forms, and telehealth approach. To ensure the LPN provided the intervention as intended, the GNP observed the LPN with her first 2 caregivers and reviewed the videos from these visits.

After the training was completed, each consultant met one-to-one with a caregiver for 8 weekly, hour-long sessions via Health Insurance Probability and Accountability Act (HIPAA)–secure, internet-based videoconferencing. The consultants followed the Tele-STAR manual, which guided them through the weekly sessions [23]. Caregivers used a workbook, which was a revision of the original STAR-C workbook [32]. The bound workbook, which contained all information, handouts, and space for writing notes, was mailed to the caregivers before session 1. Caregivers were encouraged to write in the workbook and keep it for later reference. At each session, caregivers were asked to read their notations to the consultant and/or show them their written work. Recognizing that shared authority is essential to adult learning, we asked caregivers to show us their written work on the video screen, but this was not a requirement for study participation [33].

At the first session, caregivers identified 2 or 3 care recipient target behaviors that they found distressing. Over the next 7 weeks, consultants guided the caregivers through a process in which they identified a plan to address the *a* ctivators of the behaviors, the *b* ehaviors, and the *c* onsequences (ABC). The consultants, following the Tele-STAR manual, provided information on communication strategies, pleasant events, and caregiver health. After the second assessment (2 months after completing the intervention), caregivers joined 1 final videoconferencing meeting in teams of 3 dubbed “Tele-STAR Trios,” to meet each other, and if they so desired, exchange contact information. All Tele-STAR sessions were recorded then stored in the HIPAA-secure cloud-based site.

**Table 1:** Tele-STAR measures.

Measure^a,b^	Description
Revised Memory and Behavior Problems Checklist [25]	24 items Documents the frequency of distressing care recipient behaviors and rates caregivers’ reactions to these behaviors Among the most commonly used measures of burden in caregiver research [26] Excellent reliability when used with telehealth technology (ICC^b^=.80) [27]
Zarit Burden Interview [28]	4 items Reliable (ICC=.79) when used with telehealth [27]
Desire to Institutionalize, Revised [24,29]	5 dichotomous items and 1 modified item that rates the likelihood of placement on a 5-point Likert scale (1: “not at all likely” to 5: “very likely”)
Quality of Life in Alzheimer’s Disease (QOLAD) [30]	13 items, option for comments
Montreal Cognitive Assessment [31]	30-point assessment that measures cognitive function that is reliable when used with telehealth (ICC=.93) [27] Used with care recipient only, if no formal assessment within 1 year of study start

^a^All data were collected on all participants because of the research assistant’s ability to connect with the participants via videoconferencing.

^b^ICC: intraclass correlation coefficient.

To foster treatment fidelity, the 2 consultants met every week to review the process and discuss challenges. The consultants met with the content experts 3 times over the course of the study to discuss the protocol and the intervention approach.

### Fidelity Procedures

We structured our fidelity assessment framework based on Griffiths et al’s (2016) [12] and Teri et al’s (2010) [20] approaches (Table 2). Like these authors, we engaged experts who were very familiar with the original protocol. The senior expert (LT), who designed the original STAR-C [15], provided assistance with developing the assessment criteria and mentoring the PI (AL). A total of 3 content experts (DL, LAM, GM) volunteered their time to assist with the development of the assessment criteria, assess the written materials, and view a sample of Tele-STAR videos. All 3 experts have extensive experience with the STAR-C program and with fidelity assessment of the program.

Before implementing Tele-STAR, criteria for program and treatment fidelity were identified and reviewed by the senior expert and content experts [15,17,20]. We then gave the content experts a packet with the materials to assess and work sheets to document their reviews. They had the option to complete the assessments either online or traditionally with paper and pencil.

To assess program fidelity, the content experts assessed 30 components of 4 Tele-STAR domains: General principles (12 components), homework and handouts (9 components), consultant documents (6 components), and Tele-STAR-specific documents (3 components). They were asked to use the worksheet to rate the materials in each domain with the following scale (1=inconsistent with STAR-C, 2=Same as STAR-C, and 3=adds to STAR-C) [12].

Content experts assessed treatment fidelity by viewing 12 videos of Tele-STAR sessions, which recorded the participants and consultants as they progressed through the protocol. The content experts were given access to the videos via to the university’s secure, HIPAA-compliant, cloud-based system for storing and sharing documents and videos. We used a store-and-forward process to provide the content experts with video recordings of a subsample of all Tele-STAR sessions [34]. The content experts accessed and viewed videos at a later date at their convenience. Using the Consultant Adherence Checklist worksheet [20], they rated how well the treatment information was provided by the consultants and enacted by the caregivers (0=not applicable, 1=not at all/some, 2=moderately, and 3=extensively). The 3 content experts each reviewed and rated the same sessions (2 and 5) for the same 2 caregivers, assessing a total of 12 videos. They then rated 7 more videos of their choice for 4 more caregiver participants. The content experts could choose from several participants but could only view sessions 2 and 5 to maintain consistency across reviews.

For each video, the content experts assessed 6 components: General homework review, behavior change planning, maintaining and focusing on current and observable behavior, assisting caregiver in developing own solutions, responsiveness to caregiver’s current needs, and overall quality of sessions. We calculated the percentage of caregivers who completed all sessions and prescribed homework.

### Data Analysis

This was a pilot study, and thus, data analyses were for exploratory purposes. Efficacy was assessed by comparing the measure scores before, during, and after the intervention using paired *t* tests [35]. As the consultants (the LPN and GNP) had different training backgrounds, we examined if there were differences in Revised Memory and Behavior Problems Checklist (RMBPC) change [25] by consultant using simple *t* tests.

For program fidelity, we calculated the percentage that each component was rated 1=inconsistent with STAR-C, 2=same as STAR-C, or 3=adds to STAR-C. We compared scores across the 3 expert consultants to identify agreement trends. We planned to use the Fleiss kappa to assess inter-rater agreement [36,37].

For treatment fidelity, we assessed consultant adherence to the weekly session content and the percent of caregivers attending 8 or more treatment sessions. We compared the proportion of content coverage for each consultant in Tele-STAR to content coverage in the original STAR-C study [15]. We calculated the percentage that each component on the Consultant Adherence Checklist was rated: 0=not applicable, 1=not at all/some, 2=moderately, 3=extensively. As assessing the videos could be subjectively interpreted, we used Cohen kappa coefficient to calculate inter-rater agreement, with the following parameters: almost perfect 0.81-1.00, substantial 0.61-0.80, moderate 0.41-0.60, fair 0.21-0.40, and slight/poor <0.00 [38].

**Table 2:** Tele-STAR fidelity assessment components.

Component	Element
Program fidelity [12]	Guiding principles: Do the materials reflect the guiding theory and principles of the original program (STAR-C)? Homework and handouts: Consistent with those used in STAR-C? Consultant documents: Align with the principles and goals of STAR-C? Tele-STAR-specific documents: Does the Tele-STAR training outline and logo follow STAR-C principles?
Treatment fidelity [20]	Was the Tele-STAR intervention delivered following the STAR-C approach?: Consultant Adherence Checklist (used with videos) Did forms reflect treatment receipt?: (1) Attendance records (% complete), (2) Content checklist (% complete), (3) Participant compliance measure (% complete)

## Results

### Participants

The majority (77%) of the caregivers and 31% of the care recipients were women, and all were white (Table 3). All of the caregivers were spouses or siblings. Care recipients were, for the most part, in the moderate stages of dementia. A third of the families lived in rural areas. Of the 13 caregivers who consented to Tele-STAR, 1 dropped out and was lost to follow-up after the second visit. This caregiver had a higher depression score, but not burden score, than the other caregivers.

The caregivers had newer computer models (1-3 years) and were moderately comfortable using telehealth videoconferencing (average 3.6; range 1: not at all comfortable to 5: extremely comfortable). The majority found Tele-STAR very convenient and, given the choice, would prefer a telehealth option over an in-home one (Table 4).

**Table 3:** Demographics (n=13; one lost to follow-up).

Participants			Mean (SD); range
**Caregivers**			
	Age	67.1 (7.4); 56-78	
	Number of years caregiving	3.4 (2.5); 0.5-9	
**Care recipients**			
	Age	71.5 (9); 56-83	
	Number of years with Alzheimer Disease and Related Dementias	3.6 (1.9); 1-7	
Montreal Cognitive Assessment			15.2 (3); 10-20
**Both^a^**			
	Miles from study center	39.1 (53.6); 1-169	

^a^Miles each dyad lived from the study.

**Table 4:** Participant experience rating (n=11; surveys emailed to caregivers; 1 did not respond).

Question and response		n (%)	
**Overall, how convenient was your Tele-STAR experience?**			
	Very convenient		11 (90)
	Somewhat convenient		1 (10)
	Not convenient		0 (0)
**I had good technical support from study team**			
	Agree		11 (90)
	Neutral		0 (0)
	Disagree		0 (0)
	No answer		1 (10)
**I could easily see and hear the consultant**			
	Agree		11 (100)
	Neutral		0 (0)
	Disagree		0 (0)
**It was easy to connect with my consultant via videoconferencing**			
	Agree		11 (100)
	Neutral		0 (0)
	Disagree		0 (0)
**If you had the option of in-home or telehealth training, which would you prefer?**			
	In-home		2 (18)
	Telehealth		3 (27)
	Combination of both		6 (55)

Caregivers contacted their own health care providers once a year or less (average 5.3; range 1-6, 1=more than once a week to 6=once a year or less). Of the 13 participants, 7 attended support groups. There were no significant differences in the RMBPC change between those who attended support groups and those who did not.

Caregivers contacted their care recipient’s health care provider about once per month (average 4.7; range 1-6). A total of 4 caregivers gave “as needed” medications daily to their family members; 10 did not give any as needed medications.

### Results: Tele-STAR

Caregivers’ depression and burden decreased (improved) slightly, but not significantly, by session 4 (Table 5). Burden improved significantly after session 8 as indicated by improvements in caregiver reactivity to upsetting behaviors, measured on the RMBPC [25]. This effect was sustained at 2 months postintervention. The frequency and reactivity to the target behaviors reduced significantly and was appreciated clinically by the caregivers. Ratings on the RMBPC did not differ significantly by consultant type (LPN vs GNP; *P*=.50 and .47, respectively).

### Results: Program Fidelity

**Figure 1:**
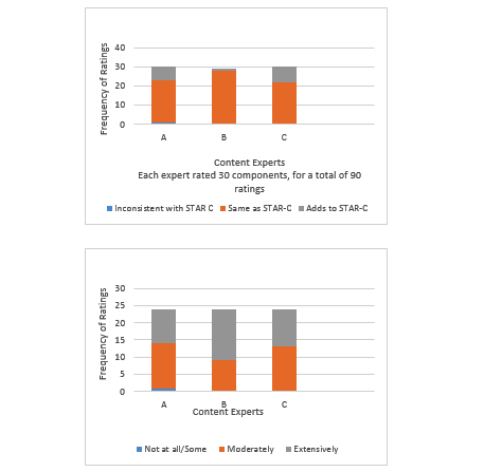
Program fidelity ratings (top) and treatment fidelity ratings (bottom).

The 3 content experts rated 30 Tele-STAR components for a total of 90 ratings (3 per component) (Figure 1, top). Of these, 72 out of the 90 ratings (80%) were labeled as “same as” STAR-C, and 16 of the 90 ratings (17%) were labeled “adds to” STAR-C. One rating was labeled “inconsistent with STAR-C,” and 1 data point was missing. There was total agreement between 3 content experts for 18 of the 30 (60%) of the components. There was some disagreement in 11 (36%) of the rated components, but the disagreement was between whether the components were “same as STAR-C” or “adds to STAR-C,” indicating 96% agreement that Tele-STAR was the same as or adds to STAR-C. Content experts noted the Tele-STAR trios “added to” the intervention. The lack of variability in the ratings made a Fleiss kappa statistic unstable. We chose not to report it because the value is not meaningful [37]. Content experts spent about 2 to 4 hours each on this assessment. These findings indicate the Tele-STAR adhered to the theoretical foundation and components of STAR-C.

**Table 5:** Tele-STAR results (n=12).

Variable^a^	Baseline, mean (SD)	Post session 4, mean (SD)^b^	Post session 8, mean (SD)	*P* value	2 months postintervention, mean (SD)^c^	*P* value^c^
CESD-10^d^	10.9 (3.4)	11.1 (4.7)	10.7 (5.5)	.88	11.8 (4.4)	.46
ZBI^e^	9.1 (2.2)	8.1 (2.5)	8.3 (2.4)	.22	8.9 (1.6)	.38
QOL-AD^f^	32.5 (7.2)	N/A^g^	33.1 (7.7)	.55	31.4 (5.5)	.37
Overall RMBPC^h^-frequency	41.8 (10.4)	N/A	41.1 (11.1)	.73	41.7 (12.6)	.81
Overall RMBPC-reactivity^i^	32.3 (12.5)	N/A	28.2 (10.4)	.04^k^	30.4 (8.9)	.41
Target symptom 1 frequency^j^	3.3 (1.1)	N/A	2.3 (1.7)	.15	—^m^	—
Target symptom 1 reactivity^k^	2.7 (0.9)	N/A	1.3 (1.1)	.0001^l^	—	—
Target symptom 2 frequency^j^	3.7 (0.5)	N/A	2.4 (1.3)	.01^l^	—	—
Target symptom 2 reactivity^k^	3.0 (0.7)	N/A	1.5 (1.2)	.001^l^	—	—

^a^Excludes baseline data for one caregiver who dropped out.

^b^Only Center for Epidemiological Studies Depression Scale-10-item (CES-D 10) and Zarit Burden Interview (ZBI) were assessed after session 4.

^c^Change from post session 8.

^d^CESD-10: Center for Epidemiological Studies Depression Scale-10-item.

^e^ZBI: Zarit Burden Interview.

^f^QOL-AD: Quality of Life-AD.

^g^N/A: not applicable.

^h^RMBPC-frequency: Revised Memory and Behavior Problems Checklist, frequency of behavioral symptoms.

^i^RMBPC-reactivity: Revised Memory and Behavior Problems Checklist, Caregiver’s reactions to behavioral symptoms.

^j^Behavioral symptoms identified by caregivers, frequency of behavioral symptoms.

^k^Caregiver’s reactions to behavioral symptoms.

^l^*P* value <.05.

^m^Target symptoms frequency and reactivity were measured only post session 8.

### Results: Treatment Fidelity

Similar to Teri et al’s findings (2005) [15] for STAR-C, 92% (12 out of 13) of the caregivers completed all Tele-STAR sessions. Similarly, 92% of the caregivers (the 12 that completed all the sessions) completed the homework assignments and developed at least one ABC plan. These findings indicate that the vast majority of the caregivers were able to understand and engage in the treatment protocol. The content experts rated consultant adherence to the protocol as “moderately” for 56 out of the 114 (49%) of the components and “extensively” for 57 of the 114 components (50%), with 1 expert rating 1 component (“Assisting Caregiver in Developing Own Solutions”) as “not all or some” for 1 video only (Figure 1, bottom). Inter-rater agreement was moderate (kappa=.43) [38]. Content experts spent about 1.5 to 2.5 hours assessing each video. These findings show that the consultants were able to follow the protocol and implement it as designed.

## Discussion

This pilot study assessed the preliminary efficacy of a revised telehealth-based intervention (Tele-STAR) and the fidelity of Tele-STAR to the original caregiver intervention (STAR-C) [15]. We found that the Tele-STAR intervention in this small sample reduced burden on the RMBPC [25] but did not improve on depression scores (Table 5). Tele-STAR had good program and treatment fidelity to STAR-C. Our fidelity assessment suggests that the Tele-STAR intervention adhered to the original STAR-C protocol and that it was implemented as designed. The implication being that the caregiver burden was reduced by the intervention and not by extraneous factors caused by divergence from the STAR-C program.

Assessing fidelity is a complex process involving time, expertise, and resources. Perepletchikova and others (2009) identified multiple barriers to fidelity assessment and found, in their review of 147 randomized trials, that only 3.5% of the papers adequately described comprehensive fidelity assessments embedded in the trials. The authors argued that to improve consumer trust, scientists need to implement, and then report, fidelity assessment findings. However, these authors also reported that fidelity assessment demands resources that many scientists lack, namely, money, time, and senior expertise. In contrast to these findings, we found that the use of videoconferencing allowed for a feasible fidelity assessment. The technological strategies modulated financial demands, maintained quality, minimized demands on content experts’ time and allowed access to nationally recognized experts.

As we had access to the university-based telehealth system that allowed video-recording of the intervention sessions, we were able to complete this assessment with minimal cost. Unlike Teri’s fidelity assessment (2010), we did not need to use direct observation strategies. If we had performed direct observation, we would have had to pay staff for the time and travel, costing a minimum of US $1500.00 [24]. None of our content experts or staff had to travel to any site for this fidelity project, making the assessment financially feasible.

Using video recordings allowed for high-quality, multidimensional assessment of the intervention sessions. The video recordings displayed participant and consultant nonverbal cues and body language [34] along with the audio. Participants consented to the recording, but the unobtrusive nature of the recording was, for the most part, ignored by the participants. Consultants were able to view the videos on their own schedules. This store-and-forward approach allowed the content experts to perform quality treatment fidelity assessments via a secure link, at their own pace. The literature supports the fidelity of assessing interactive sessions using store-and-forward strategies. An Agency for Healthcare Research and Quality study found that telehealth fidelity assessments work best when there is a high degree of human interaction (as in the Tele-STAR intervention) and less so when the activity involves complex diagnostic testing [34]. This ability to record the sessions speaks to a maturation of technology that can facilitate fidelity assessment at a lower cost, while at the same time retain quality.

Our study provided a unique opportunity to evaluate fidelity with a program (STAR-C) [15] that had undergone previous fidelity assessments [20]. Thus, we had published guidelines and skilled experts to facilitate the process. Further, even though some of the experts lived in other states, the technology allowed for content expert assessment and team meetings, minimizing time demands for all.

The videoconferencing technology not only allowed for implementation of the intervention, it provided a medium for expert access, indicating that content experts do not need to be on-site to provide valuable feedback and guidance. In our case, we were able to engage remote content experts who were intimately familiar with the earth-bound STAR-C program [15]. Their deep knowledge of the theoretical framework, the applied intervention, and the appropriate expectations for the consultants allowed for sophisticated mentoring for the PI (AL), skilled assessment of fidelity, and ready feedback for consultants in a timely manner.

Taken together, the usable technology, expert guidance, and published protocols facilitated a fidelity assessment that was informative and laid the groundwork for future assessments. Through this process, the limitations to our pilot study were highlighted. Specifically, the ordinal assessment scales prevented sophisticated statistical analyses for the fidelity assessment. Also of concern is the racial homogeneity of the sample. Although we were able to recruit underrepresented rural participants, we fell short of our goals in recruiting African American and Asian participants. Despite these limitations, we learned that our intervention adhered to the STAR-C [15] intervention and was effective in reducing caregiver burden.

Embarking on a fidelity assessment can be nerve-wracking for scientists. Researchers may be reluctant to perform a fidelity assessment because the assessment may identify poor fidelity to the protocols, which can undermine the scientific credibility [39]. This concern speaks to the need to include fidelity assessment in the pilot phases of early-stage research [19] and to engage experts in process. Our pilot study allowed for testing of our fidelity assessment, identified areas for improvement, and laid the foundation for more sophisticated assessments of future iterations of Tele-STAR.

As the use of technology for caregiver support expands, so does the risk for interventions to become detached from the original protocols. Yet, technology also allows for creative solutions that can both change an intervention or improve upon it. In this rapidly evolving world, tactics to maintain fidelity are essential to long-term programmatic success.

## Abbreviations

ABC: activators of the behaviors, the behaviors, and the consequences
GNP: gerontological nurse practitioner
HIPAA: Health Insurance Probability and Accountability Act
LPN: licensed practical nurse
OHSU: Oregon Health and Science University
PI: principal investigator
RMBPC: Revised Memory and Behavior Problems Checklist
