# Table Correction: Using Technology to Facilitate Fidelity Assessments: The Tele-STAR Caregiver Intervention

**DOI:** 10.2196/18032

**Published:** 2020-04-28

**Authors:** Allison Lindauer, Glenise McKenzie, David LaFazia, Loriann McNeill, Kate Mincks, Natasha Spoden, Marcella Myers, Nora Mattek, Linda L Teri

**Affiliations:** 1 Layton Aging and Alzheimer's Disease Center Oregon Health & Science University Portland, OR United States; 2 School of Nursing Oregon Health & Science University Portland, OR United States; 3 School of Social Work University of Washington Seattle, WA United States; 4 Northwest Research Group on Aging School of Nursing University of Washington Seattle, WA United States; 5 Family Caregiver Support Program Multnomah County, Oregon Portland, OR United States; 6 Northwest Roybal Center School of Nursing University of Washington Seattle, WA United States

In the original published paper “Using Technology to Facilitate Fidelity Assessments: The Tele-STAR Caregiver Intervention” (J Med Internet Res 2019; 21(5):e13599), the authors noticed an error in Table 5. This was caused by a coding error on the authors' electronic survey. The original table listed the 10-item Center for Epidemiological Studies Depression Scale (CESD-10) score for the time point “Post session 4” lower than it should have been.

The score was initially listed as:

Mean 9.4, SD 6.8 (row 1, column 3), *P*=.84 (row 1, column 5).

The correct score is:

Mean 11.1, SD 4.7 (row 1, column 3), *P*=.88 (row 1, column 5).

The changes were not significant, and do not affect the overall findings of the paper.

The correction will appear in the online version of the paper on the JMIR website on April 28, 2020, together with the publication of this correction notice. Because this was made after submission to PubMed, PubMed Central, and other full-text repositories, the corrected article has also been resubmitted to those repositories.

